# Endothelial function and vascular events in patients with limited cutaneous systemic sclerosis (EFVELSS): a prospective observational study

**DOI:** 10.1007/s00296-025-05919-y

**Published:** 2025-07-08

**Authors:** Philipp Jud, Philipp Douschan, Teresa Sassmann, Günther Silbernagel, Katharina Gütl, Reinhard B. Raggam, Peter Rief, Leyla Schweiger, Nikolaus John, Gerald Seinost, Vasile Foris, Gabor Kovacs, Thomas Gary, Viktoria Nemecz, Andreas Meinitzer, Heimo Strohmaier, Balazs Odler, Florentine Moazedi-Fürst, Marianne Brodmann, Franz Hafner

**Affiliations:** 1 Division of Angiology, Department of Internal Medicine, Medical University of Graz, Auenbruggerplatz 15, Graz, 8036 Austria; 2 Division of Pulmonology, Department of Internal Medicine, Medical University of Graz, Graz, Austria; 3 Ludwig Boltzmann Institute for Lung Vascular Research, Graz, Austria; 4 Channing Division of Network Medicine, Department of Medicine, Brigham and Women´s Hospital, Harvard Medical School, Boston, MA USA; 5 Clinical Institute of Medical and Chemical Laboratory Diagnostics, Medical University of Graz, Graz, Austria; 6 Center of Medical Research (ZMF), Medical University of Graz, Graz, Austria; 7 Division of Nephrology, Department of Internal Medicine, Medical University of Graz, Graz, Austria; 8 Division of Rheumatology, Department of Internal Medicine, Medical University of Graz, Graz, Austria

**Keywords:** Endothelium, Interstitial lung disease, Microcirculation, Pulmonary hypertension, Systemic sclerosis

## Abstract

Evaluating the predictive role of endothelial dysfunction in the development of vasculopathy-mediated complications in patients with limited cutaneous systemic sclerosis (lcSSc). 38 patients with lcSSc who were naïve for vasculopathy-mediated complications, defined as absent pre-existing digital ulcers (DU), pulmonary hypertension (PH) and symptomatic atherosclerotic cardiovascular diseases, were prospectively observed during a 3-years follow-up period. At study enrolment, functional and laboratory parameters of endothelial dysfunction were assessed. Microvascular events, defined as the development of DU and PH, macrovascular events, defined as newly symptomatic atherosclerotic cardiovascular diseases, and clinical events, defined as additional interstitial lung disease (ILD), renal crisis and esophageal dysfunction, were recorded annually. 33 patients (86.8%) completed all follow-up study visits. During the follow-up period, DU, PH, carotid and vertebral artery disease and ILD occurred in eight (24.2%), one (3.0%), one (3.0%) and two patients (6.1%), respectively, without recorded renal crisis and esophageal dysfunction. Number of pathologic FMD, NMD and/or PWV values was significantly associated with the development of clinical events (*p* = 0.035), but was not a significant predictor for microvascular events. Number of pathologic FMD, NMD and/or PWV values was a significant predictor for disease activity assessed by EUSTAR index ≥ 2.5 at last study visit (OR 5.47 [95% CI 1.01–29.03], *p* = 0.049). Distinct endothelial dysfunction may promote the development of disease complications and disease activity in patients with lcSSc.

**Supplementary Information:**

The online version contains supplementary material available at 10.1007/s00296-025-05919-y.

## Introduction

Systemic sclerosis (SSc) is a systemic autoimmune connective tissue disease characterized by vasculopathy and progressive fibrosis of the skin and various internal organs. In SSc, functional and structural changes affecting predominately microvasculature may occur due to complex interactions between different cell types and autoimmune activation, while limited cutaneous systemic sclerosis (lcSSc) may especially be accompanied by vasculopathy [1, 2]. Those changes may contribute to clinically relevant symptoms and complications of lcSSc, like Raynaud’s phenomenon, digital ulcer (DU) or pulmonary hypertension (PH) [2, 3]. Additionally, potential associations of vasculopathy in renal and gastrointestinal involvement in SSc have been described [4, 5]. In addition, several studies reported macrovascular changes in patients with SSc such as having a higher risk for cardiovascular diseases, including myocardial infarction or stroke [6, 7]. Furthermore, functional parameters of macrovascular involvement, like flow-mediated dilation (FMD) or pulse-wave velocity (PWV), are significantly altered in SSc patients [7, 8]. Besides those functional parameters, there are several other biomarkers of endothelial dysfunction, including parameters of the arginine metabolism, endothelial microparticles (EMP) or von Willebrand factor (vWF), which have been investigated in SSc and were associated with disease complications [9–11].

Although various studies investigated endothelial dysfunction in SSc, they have some limitations. Several studies included either SSc subjects with experienced vasculopathy-mediated complications, like DU, PH or cardiovascular diseases, or a combined SSc subject population with lcSSc and diffuse cutaneous SSc, despite pathogenetic differences between those phenotypes [9–13]. Moreover, prospective studies and data about the predictive value of endothelial dysfunction are limited.

Therefore, the aim of this study was to investigate prospectively the incidence of disease-specific complications and cardiovascular disease in lcSSc patients, who were naïve for vasculopathy-mediated complications, its association with endothelial dysfunction and changes of lcSSc-specific parameters during a 3-year observational follow-up period.

## Materials and methods

### Study design and patient cohort

**Fig. 1:**
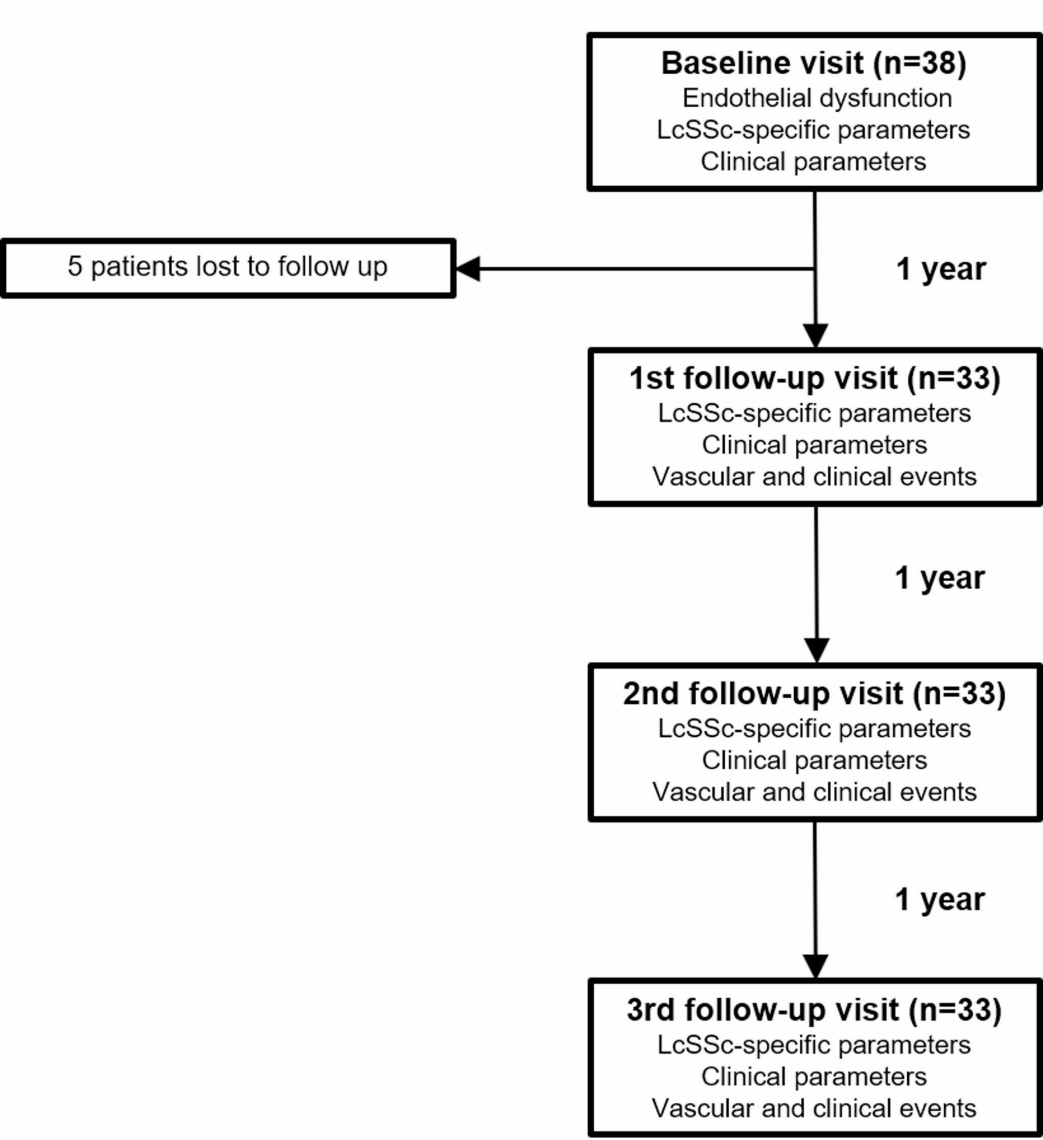
Flow chart of the study assessments during follow-up period

This study is an observational follow-up study of patients involved in a previously published study, which investigated endothelial dysfunction in patients with lcSSc without known DU, PH and symptomatic atherosclerotic cardiovascular diseases. Full details about parameter measurements have been previously described [14]. The follow-up period was 3 years. Baseline study visit was performed between April 2019 and February 2020 with subsequent annual (± three months) follow-up study visits. Parameters of endothelial dysfunction, lcSSc-specific and clinical parameters (as detailed below) were evaluated at baseline visit. LcSSc-specific and clinical parameters as well as the development of lcSSc-specific disease complications and cardiovascular disease (called as vascular and clinical events) were recorded at annual study visits (Fig. 1). This study was finished in May 2023.

Patients with lcSSc who fulfilled classification criteria according to the EULAR/ACR criteria of 2013 were included [15]. Exclusion criteria at study enrolment were age < 18 years, presence of diffuse cutaneous SSc or other connective tissues diseases, preexisting or existing DU, PH, endoscopic proven reflux, diabetes mellitus or symptomatic atherosclerotic cardiovascular diseases, recent pregnancy or malignancies, acute infections at time of enrolment and current intake (< 24 h) of prostanoids, calcium channel blockers, phosphodiesterase-5 inhibitors or endothelin-receptor inhibitors.

Primary endpoint was the association between endothelial dysfunction and the incidence of vascular and clinical events. Secondary endpoints were associations between endothelial dysfunction and lcSSc-specific parameters during follow-up period.

### Evaluation of endothelial dysfunction

Functional and laboratory parameters of endothelial dysfunction were assessed. Functional parameters of endothelial dysfunction included FMD, nitroglycerine-mediated dilation (NMD), PWV and augmentation index (Aix). Vascular reactivity was measured by FMD and NMD according to the guidelines by Corretti et al. [16] while arterial stiffness was measured by PWV and Aix with an oscillometric device (I.E.M. Mobil-O-Graph, I.E.M., Stolberg, Germany) using automated pulse-wave analysis. FMD < 7%, NMD < 15.6% and PWV > 10 m/s were defined as pathologic [17–19]. Patients were categorized into four groups if none, one, two or all three values of FMD, NMD and/or PWV were pathologic. Fasting blood samples were obtained for the assessment of laboratory parameters of endothelial dysfunction, including asymmetric dimethylarginine (ADMA), symmetric dimethylarginine (SDMA), homoarginine, arginine, CD31+/CD42b− EMP, and vWF antigen (vWF: Ag) and activity (vWF: Ac). Parameters of the arginine metabolism were assessed by high-performance liquid chromatography [20, 21]. EMP were measured according to the recommendations published by Cossarizza et al. [22]. Plasma vWF: Ag and vWF: Ac were measured in the same laboratory. Detailed information about the measurement methods of the respective parameters of endothelial dysfunction have been previously published [14].

### Vascular and clinical events

Vascular events were divided into microvascular and macrovascular events. Microvascular events were defined as development of DU and PH, while macrovascular events included newly-developed symptomatic coronary heart disease (CAD), carotid and vertebral artery disease (CVAD), and peripheral artery disease (PAD). Symptomatic CAD included new-onset acute coronary syndrome, symptomatic CVAD included new-onset stroke or transient ischemic attack and symptomatic PAD was defined as new-onset of claudication, rest pain or ischemic gangrene according to Fontaine stage II-IV. Clinical events encompassed all vascular events including additionally the development of interstitial lung disease (ILD), renal crisis and esophageal dysfunction. DU were recorded using patients’ history and clinical charts as well as by physical examination. PH was recorded using patients’ history and clinical charts as well as by standardized screening transthoracic echocardiography with potential subsequent right heart catheterization using a standardized protocol for the measurement of hemodynamic values at the PH center of the division of pulmonology. PH was defined as mean pulmonary arterial pressure > 20 mmHg, pulmonary vascular resistance > 2WU and pulmonary arterial wedge pressure ≤ 15 mmHg [23]. Symptomatic CAD, CVAD and PAD were recorded using patients’ history and clinical charts. ILD was recorded using patients’ history and clinical charts as well as by screening spirometry including bodyplethysmography and diffusing capacity of the lung for carbon monoxide (DLCO) with potential subsequent high-resolution computed tomography of the lungs. ILD diagnosis was established based on a decision by an interdisciplinary ILD board consisting of pulmonologists, radiologists, and rheumatologists. History on renal crisis was recorded using patients’ history and clinical charts and was defined as an abrupt onset of severe arterial hypertension and rapidly progressive kidney failure based on changes in estimated glomerular filtration rate (eGFR) and urine total protein/creatinine ratio. Esophageal dysfunction was recorded using patients’ history and clinical charts as well as by UCLA SCTC GIT 2.0 total score with potential subsequent endoscopy, esophagogram or manometry.

### LcSSc-specific and clinical parameters

Telangiectasia, puffy finger, sclerodactyly, modified Rodnan Skin Score (mRSS), UCLA SCTC GIT 2.0 total and constipation score, capillaroscopic skin ulcer risk index (CSURI), early, active and late pattern, DETECT score, and EUSTAR index were defined as lcSSc-specific parameters. Interobserver variability for telangiectasia was 0.92, for puffy finger 0.82, for sclerodactyly 0.84, for mRSS 0.69, for CSURI 0.46, for early pattern 0.59, for active pattern 0.52 and for late pattern 0.55. Prevalent telangiectasia, puffy finger, sclerodactyly and mRSS were recorded by physical examination and UCLA SCTC GIT 2.0 total and constipation score by respective questionnaire [24]. CSURI, early, active and late pattern were recorded by nailfold videocapillaroscopy of the second to fifth digit on both hands (Skinview, Optometron Ltd., Oskar-Messterstr., Ismaning, Germany) [25]. DETECT score and EUSTAR index were recorded according to published data [26, 27].

C-reactive protein (CRP), eGFR based on the CKD-EPI equation, urine total protein/creatinine ratio, N-terminal prohormone of brain natriuretic peptide (NT-proBNP), predicted DLCO, FEV1%FVC and total lung capacity (TLC) were defined as clinical parameters and were measured by laboratory analysis, spirometry and DLCO.

### Sample size calculation

Since this study is a follow-up study of a previous published study, separate sample size calculation for the recent study was not performed, but has been conducted for the previous published study [14].

### Statistical analysis

Continuous variables were represented as mean and standard deviation or median and interquartile range. Categorical variables were represented by frequency and percentages. Normal distribution was examined with Kolmogorov-Smirnov test. Interobserver variability of lcSSc specific parameters, including clinical changes, mRSS, CSURI, and capillaroscopic pattern was represented by Cohen’s kappa. Chi-square test was used analyzing categorical variables, eta coefficient with subsequent ANOVA was utilized analyzing categorical with metric variables, and paired sample t-test was used analyzing differences of matched samples. Pearson’s correlation coefficient was used for normally distributed variables and Spearman’s correlation coefficient was used for non-normally distributed variables. Univariate linear and logistic regression analysis were performed to assess parameters of endothelial dysfunction as predictors for vascular and clinical events and for changes in lcSSc-specific parameters. Due to the small sample size given an exploratory study design, statistical analysis were unadjusted for multiple testing. Statistical significance was assumed for p-values < 0.05 and statistical analyses were executed with SPSS version 29.0.

### Ethics approval

This study was approved by the local ethics committee (protocol number EK 29–361 ex 16/17) and was conducted in accordance with the Helsinki Declaration of 1975, as revised in 2013. All patients gave their written informed consent after accurate information about the study.

## Results

Thirty-three patients (86.8%) completed all follow-up study visits while five patients (13.2%) were lost to follow-up, as they refused further study visits during the COVID-19 pandemic in 2020/2021. Latter patients were excluded from statistical analysis including baseline measurements. Patients characteristics and parameters of endothelial dysfunction at baseline measurement are listed in Table 1..

**Table 1.** Patients’ characteristics and parameters of endothelial dysfunction at baseline visit

	LcSSc (n=33)
Age (years), mean (± SD)	57.45 ± 9.47
BMI, mean (± SD)	23.62 ± 3.51
Disease duration (years), median (25^th^−75^th^ percentiles)	5.6 (2.8-11)
Sex, n (%)	
Female	32 (97.0)
Male	1 (3.0)
Antinuclear antibodies, n (%)	31 (93.9)
Elevated anti-centromere antibodies against centromeric protein B	23 (69.7)
Elevated anti-topoisomerase I antibodies	3 (9.1)
Cardiovascular risk factors, n (%)	
Arterial hypertension	11 (33.3)
Hyperlipidemia	17 (51.5)
Obesity	3 (9.1)
Active smoking	4 (12.1)
Ex-smoking	7 (21.2)
Medication, n (%)	
ACE inhibitors/ARB	7 (21.2)
Beta-Blocker	2 (6.1)
Calcium channel blockers	5 (15.2)
Diuretics	1 (3.0)
Anticoagulants	2 (6.1)
Platelet aggregation inhibitors	6 (18.2)
Statins	3 (9.1)
Immunosuppressive agents	5 (15.2)
Abatacept	1 (3.0)
Hydroxychloroquine	2 (6.1)
Methotrexate	1 (3.0)
Mycophenolate mofetil	1 (3.0)
Prednisolone	3 (9.1)
Number of pathologic FMD, NMD and/or PWV values, n (%)	
None	6 (18.2)
One	21 (63.6)
Two	6 (18.2)
Three	0 (0.0)
FMD (%), median (25-75^th^ percentile)	3.23 (1.30-6.67)
FMD < 7%, n (%)	26 (78.8)
NMD (%), median (25-75^th^ percentile)	19.80 (15.99-22.84)
NMD < 15.6%, n (%)	4 (12.1)
PWV (m/s), mean (± SD)	8.16 ± 1.56
PWV >10 m/s, n (%)	4 (12.1)
Aix, mean (± SD)	26.18 ± 13.53
ADMA (μmol/L), mean (± SD)	0.66 ± 0.11
SDMA (μmol/L), mean (± SD)	0.68 ± 0.15
Arginine (μmol/L), mean (± SD)	113.00 ± 14.46
Homoarginine (μmol/L), mean (± SD)	1.66 ± 0.58
CD31+/CD42b- EMP (U/µl), median (25-75^th^ percentile)	29 (21-42)
vWF:Ag (%), mean (± SD)	118.3 ± 19.2
vWF:Ac (%), median (25-75^th^ percentile)	118 (108-146)

Abbreviations: *ACE* angiotensin-converting enzyme, *ADMA* asymmetric dimethylarginine, *Aix* augmentation index, *ARB *angiotensin receptor blocker, *BMI* body mass index, *EMP *endothelial microparticles, *FMD* flow-mediated dilation, *ILD* interstitial lung disease, *lcSSc *limited cutaneous systemic sclerosis, *NMD *nitroglycerine-mediated dilation, *PWV* pulse-wave velocity, *SDMA* symmetric dimethylarginine, *vWF:Ac*; von Willebrand factor activity, *vWF:Ag *von Willebrand factor antigen

### Incidence of vascular and clinical events during follow-up

During the 3-year follow-up period, DU and PH occurred in eight (24.2%) patients and in one patient (3.0%), respectively. All eight patients have received an administration of bosentan and two patients received additionally prostaglandins due to DU. A single patient with PH developed an exercise PH without initiation of vasodilation therapy. One patient (3.0%) developed symptomatic CVAD as a transient ischemic attack, while no symptomatic CAD or PAD occurred. Two patients (6.1%) developed subclinical ILD and no patient developed esophageal dysfunction or renal crisis. Median number of microvascular events per patient was 0 (min-max 0–1), median number of macrovascular events per patient was 0 (min-max 0–1), and median number of clinical events per patient was 0 (min-max 0–2).

### Changes of lcSSc-specific and clinical parameters during follow-up

**Fig. 2:**
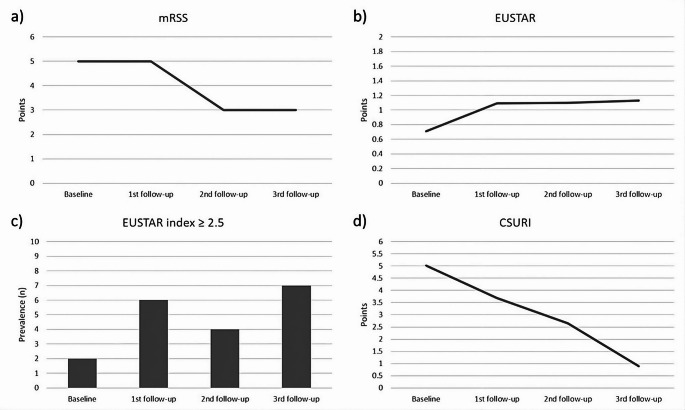
Changes of mRSS (**a**), EUSTAR index (**b**), EUSTAR index ≥ 2.5 (**c**) and CSURI (**d**) during follow-up period

**Fig. 3:**
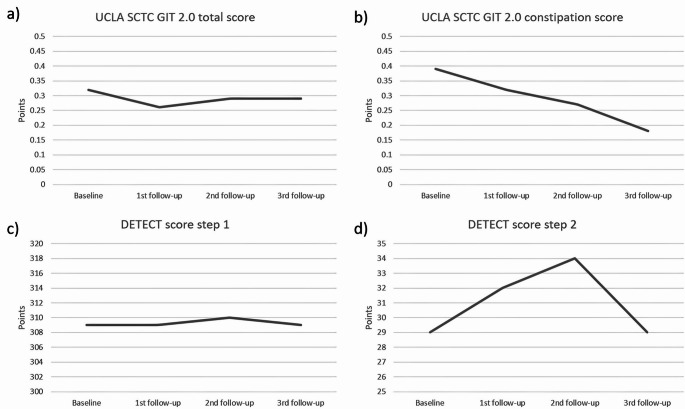
Changes of UCLA SCTC GIT 2.0 total (**a**) and constipation score (**b**), DETECT score step 1 (**c**) and step 2 (**d**) during follow-up period

Prevalence of sclerodactyly significantly decreased during the follow-up period (*p* = 0.030) without significant changes for prevalent telangiectasias and puffy finger (*p* = 0.423 and *p* = 0.475, respectively). Mean CSURI and prevalent active pattern significantly decreased during the follow-up period (*p* = 0.019 and *p* = 0.011, respectively) without significant changes for prevalent early and late pattern (*p* > 0.999 and *p* = 0.264, respectively). Mean UCLA SCTC GIT 2.0 constipation score also significantly decreased (*p* = 0.045), while mean mRSS, UCLA SCTC GIT 2.0 total score, DETECT score step 1 and 2 did not significantly change during the follow-up period (*p* = 0.059; *p* = 0.551; *p* = 0.834; *p* > 0.999, respectively). Mean EUSTAR index significantly increased during the follow-up period (*p* = 0.049), while the frequency of EUSTAR index ≥ 2.5 did not significantly increase (*p* = 0.057). Changes of telangiectasias, sclerodactyly, puffy finger, early, active and late pattern during follow-up period are depicted in supplemental Fig. 1a***–***f. Changes of mRSS, EUSTAR index, EUSTAR index ≥ 2.5 and CSURI as well as UCLA SCTC GIT 2.0 total, UCLA SCTC GIT 2.0 constipation score, DETECT score step 1 and 2 during follow-up period are depicted in Figs. 2 and 3.

Changes of clinical parameters during follow-up are depicted in supplemental Fig. 2a***–***f. FEV1/FVC significantly increased during follow-up period (*p* < 0.001) while no significant changes for CRP, eGFR, protein/creatinine ratio, DLCO and TLC during follow-up period were found (*p* = 0.240; *p* = 0.324; *p* = 0.415; *p* = 0.099; *p* = 0.513, respectively).

### Association of endothelial dysfunction with vascular and clinical events

Number of pathologic FMD, NMD and/or PWV values was significantly associated with the development of any clinical event (*p* = 0.035), but not with microvascular events (*p* = 0.064). Since there was only one event of CVAD without further macrovascular events, no appropriate statistical analysis was performed. No further associations between other parameters of endothelial dysfunction and the development of vascular or clinical events were found (Table 2).

**Table 2:** Associations between development of vascular and clinical events and endothelial dysfunction

	Microvascular events	Clinical events
Number of pathologic FMD, NMD and/or PWV values	0.064	**0.035**
FMD	0.202	0.264
FMD < 7%	0.294	0.230
NMD	0.085	0.351
NMD < 15.6%	0.385	0.174
PWV	0.057	0.370
PWV > 10 m/s	0.351	0.596
Aix	0.117	0.339
ADMA	0.458	0.344
SDMA	0.268	0.494
Arginine	0.232	0.167
Homoarginine	0.271	0.218
CD31+/CD42b- EMP	0.768	0.195
vWF: Ag	0.383	0.183
vWF: Ac	0.497	0.121

Abbreviations: *ADMA* asymmetric dimethylarginine, *Aix* augmentation index, *EMP* endothelial microparticles, *FMD *flow-mediated dilation, *NMD* nitroglycerine-mediated dilation, *PWV* pulse-wave velocity, *SDMA* symmetric dimethylarginine, *vWF: Ac* von Willebrand factor activity, *vWF: Ag* von Willebrand factor antigen. Bold values indicate statistical significance (p<0.05).

In univariate logistic regression analysis, number of pathologic FMD, NMD and/or PWV values was neither a significant predictor for the development of microvascular events (OR 3.76 [95% CI 0.88–16.11], *p* = 0.074) nor for the development of clinical events (OR 3.50 [95% CI 0.86–14.30], *p* = 0.082). Due to the low number of new macrovascular events during follow-up, no appropriate regression analysis was conducted for respective parameter.

### Associations of endothelial dysfunction and lcSSc-specific parameters

Number of pathologic FMD, NMD and/or PWV values was associated with EUSTAR index ≥ 2.5 (*p* = 0.039) at last study visit, but no associations with telangiectasias, sclerodactyly, puffy finger, early, active or late pattern were observed. Furthermore, no associations between FMD < 7%, NMD < 15.6% or PWV > 10 m/s with EUSTAR index ≥ 2.5, telangiectasias, sclerodactyly, puffy finger, early, active or late pattern at last study visit were found (all with *p* > 0.05). However, at last study visit, significant positive correlations were found between ADMA and DETECT score step 1 and 2 (*p* = 0.003 and *p* = 0.008, respectively), between SDMA and DETECT score step 1 and 2 (*p* = 0.014 and *p* = 0.010, respectively) and between homoarginine and CSURI at last study visit (*p* = 0.015). Significant negative correlations were found between NMD and EUSTAR index (*p* = 0.026) as well as between vWF: Ag and UCLA SCTC constipation score at last study visit (*p* = 0.036) (Table 3).

**Table 3:** Correlations between lcSSc-specific parameters at last study visit and endothelial dysfunction at baseline visit

		CSURI	mRSS	UCLA SCTC GIT total score	UCLA SCTC GIT constipation score	DETECT step 1	DETECT step 2	EUSTAR index
FMD	r	0.175	− 0.226	0.150	0.140	0.334	0.301	− 0.107
	p	0.372	0.231	0.429	0.459	0.103	0.111	0.562
NMD	r	− 0.271	− 0.379	− 0.122	0.101	0.010	0.281	**− 0.428**
	p	0.201	0.051	0.553	0.622	0.964	0.149	**0.026**
PWV	r	− 0.230	0.115	0.068	− 0.032	0.234	0.262	0.200
	p	0.238	0.544	0.721	0.868	0.261	0.210	0.272
Aix	r	− 0.167	0.110	0.193	0.097	0.053	0.046	0.027
	p	0.397	0.564	0.308	0.611	0.800	0.861	0.883
ADMA	r	0.136	0.068	− 0.222	− 0.075	**0.574**	**0.532**	0.324
	p	0.491	0.721	0.238	0.695	**0.003**	**0.008**	0.071
SDMA	r	− 0.034	− 0.099	0.086	− 0.021	**0.485**	**0.516**	0.234
	p	0.863	0.603	0.651	0.911	**0.014**	**0.010**	0.197
Arginine	r	− 0.284	− 0.141	0.051	− 0.049	0.089	0.271	0.077
	p	0.144	0.457	0.787	0.796	0.672	0.152	0.675
Homoarginine	r	**0.456**	0.124	− 0.167	− 0.127	0.039	− 0.064	0.044
	p	**0.015**	0.512	0.379	0.504	0.853	0.806	0.810
CD31+/CD42b- EMP	r	0.365	0.048	− 0.202	− 0.111	0.334	0.126	0.125
	p	0.056	0.799	0.283	0.560	0.103	0.630	0.495
vWF: Ag	r	− 0.342	− 0.130	0.099	**− 0.392**	0.157	0.195	− 0.217
	p	0.081	0.501	0.608	**0.036**	0.464	0.470	0.240
vWF: Ac	r	− 0.288	− 0.185	0.045	− 0.215	− 0.046	−0.043	− 0.353
	p	0.146	0.337	0.815	0.263	0.832	0.874	0.052

Abbreviations: *ADMA* asymmetric dimethylarginine, *Aix* augmentation index, *CSURI* capillaroscopic skin ulcer risk score, *DU *digital ulcers, *EMP* endothelial microparticles, *FMD* flow-mediated dilation, *mRSS* modified Rodnan skin score, *NMD* nitroglycerine-mediated dilation, *PWV* pulse-wave velocity, *SDMA* symmetric dimethylarginine, *vWF: Ac* von Willebrand factor activity, *vWF: Ag *von Willebrand factor antigen. Bold values indicate statistical significance (p<0.05).

In univariate logistic regression analysis, number of pathologic FMD, NMD and/or PWV values was significantly associated with EUSTAR index ≥ 2.5 at last study visit (OR 5.47 [95% CI 1.01–29.03], *p* = 0.049). Using univariate linear regression analysis, significant associations between homoarginine and CSURI at last study visit (*β* = 0.502, *p* = 0.006) as well as between vWF: Ag and UCLA SCTC constipation score at last study visit (*β *= − 0.376, *p* = 0.044) were found. NMD was not significantly associated with EUSTAR index at last study visit (β = − 0.343, *p* = 0.080).

## Discussion

The present study demonstrated that a distinct endothelial dysfunction pattern may be a potential contributor for the development of SSc-specific disease complications and predictor for disease activity in patients with lcSSc without pre-existing vasculopathy-mediated complications. Endothelial dysfunction encompasses an extensive spectrum of structural and functional changes of macro- and microvasculature in different entities, including inflammatory diseases, which can be measured by numerous parameters [28, 29]. In SSc, many different parameters of endothelial dysfunction, especially functional parameters such as FMD or PWV, have been described to be associated with SSc-specific changes suggesting that endothelial dysfunction plays an important role in SSc-vasculopathy [7–14]. However, most of those studies were no long-term prospective studies and did not evaluate the predictive role of endothelial dysfunction in lcSSc.

In the present lcSSc cohort, parameters of endothelial dysfunction were commonly altered at baseline, as 81.8% of all patients had at least one pathologic value either of FMD, NMD or PWV. Despite a high number of pathologic parameters of endothelial dysfunction at baseline, only the composite parameter defined as the number of pathologic FMD, NMD and/or PWV values was significantly associated with clinical events, but not each single parameter. No statistical significance was achieved for this composite parameter in simple regression analysis predicting microvascular or clinical events. On the other hand, it was a significant predictor for disease activity assessed by EUSTAR index. However, due to the low sample size and the low statistical power in our study and due to the fact that only the combination of pathologic FMD, NMD and/or PWV values into some sort of categorical assessment achieved statistical significance, this finding should be interpreted with caution. Although endothelial dysfunction might play a role on the underlying disease pathways in lcSSc, especially on vasculopathy, its predictive role for disease complications seems yet to be marginally. It seems that different pathways of endothelial dysfunction are altered in lcSSc. However, the entirety and the mutual interactions of those pathways seem to contribute more on clinical changes in lcSSc than one single parameter. Therefore, it seems that a single parameter of endothelial dysfunction may be inappropriately suited for the prediction of disease complications or disease activity in lcSSc. Although an assessment of more than one parameter of endothelial dysfunction might be feasible in patients with lcSSc, who are in an early phase of their disease or naïve for vasculopathy-mediated complications, the predictive value of parameters of endothelial dysfunction needs to be more thoroughly evaluated in larger prospective studies and also for other SSc subtypes. Especially, studies need to investigate if potential high-risk patients may profit from a closer monitoring of parameters of endothelial dysfunction to detect SSc-specific complications in an early phase. Importantly, only a low number of specific cut-off values of endothelial dysfunction, like for FMD, NMD and PWV, has been proposed or validated so far [17–19, 21]. Therefore, further studies are needed to evaluate those proposed cut-off values in large, prospectively followed patient cohorts.

This study could also demonstrate that lcSSc patients were more likely to develop vascular SSc-complications, such as DU, than non-vascular SSc-complications, like ILD, during the follow-up period. Additionally, no renal crisis or esophageal dysfunction were observed. This finding is in accordance with a recent meta-analysis reporting that specific SSc-complications occur more likely than others [30]. Additionally, there are data indicating that SSc-mediated ILD may be partially accompanied also by vasculopathy making potential contribution by endothelial dysfunction possible [31]. However, the impact of endothelial dysfunction on microvascular and macrovascular complications seems to be again marginally as no parameter of endothelial dysfunction was associated with the development of microvascular events, including DU, in the recent study although DU is commonly described as a vasculopathy-mediated complication in SSc. That absent association is in accordance with previous studies, since FMD and ADMA have been described to be potentially associated with DU, but with divergent results, and PWV revealed no associations with DU yet [32, 33]. One possible explanation may be that subclinical endothelial dysfunction is an initial trigger for vascular changes leading to DU but the promoting pathways afterwards occur rather independently from endothelial dysfunction. This hypothesis may be emphasized by our finding that only the composite parameter defined as the number of pathologic FMD, NMD and/or PWV values was significantly associated with a EUSTAR index ≥ 2.5 at last study visit, but any further parameter of endothelial dysfunction did not achieved statistical significance. This suggest that endothelial dysfunction may have potential, but only slightly predictive features for disease activity in lcSSc. Interestingly, several SSc-specific parameters decreased during follow-up, including CSURI or active pattern which are both vasculopathy-specific SSc-parameters. Those changes may be influenced on the one hand by administered drugs during the follow-up period, like immunosuppressive agents, bosentan or prostaglandins which may improve clinical changes in SSc [34, 35]. On the other hand, potential interobserver variability might also has affected SSc-specific parameters. In correlation analysis, selected parameters of endothelial dysfunction also tended to be rather correlated with vasculopathy-specific SSc-parameters, like DETECT score or CSURI, than with non-vasculopathy-specific SSc-parameters at last study visit, which was demonstrated previously [9, 36]. Consequently, endothelial dysfunction may rather be associated with typical vasculopathy-specific SSc-parameters. However, due to complex interactions with inflammatory and fibrotic pathways, its significance with selected disease complications of SSc needs to be further elucidated in larger studies.

There are several limitations of this study. The two major limitations are the small sample size and the lack of a control group. Both major limitations derive from the study design, which was designed for a previously published study and adopted for the recent observational study [14]. The sample size was calculated for a previously published study from which this exploratory study has been emerged for hypothesis generation. Additionally, a comparison group of patients with diffuse SSc or with pre-existing vasculopathy-mediated complications evaluating potential differences of endothelial dysfunction and of disease complications was missing due to the study design of the previously published study, which did not include another comparative SSc group. Due to the small sample size, only basic statistical analyses could be appropriately performed, which limit the statistical power of the recent study. Given its exploratory character of the recent study and due to the lack of a control group limits the generalizability and data interpretation of this study further. Additionally, potential interobserver variability cannot be excluded, especially for some lcSSc-specific parameters like capillaroscopic patterns or sclerodactyly, and the incidence of some vascular and clinical events, like for PH or ILD, were not prespecified in the recent study while definitions have changed during the observational period, like for PH [37]. Therefore, it may be possible that vascular and clinical events were recorded although recent definitions of the respective event were not fulfilled. Moreover, there was some kind of lacking in objective measurements of vascular and clinical events, as the incidence of SSc-specific complications were mostly recorded using patients’ history and chart review while only patients with suspected clinically relevant SSc-specific complications have undergone specific additional diagnostic work-up. Subclinical forms of SSc-specific complications, which occur in up to one third of SSc patients, were therefore not recorded since high-resolution computed tomography or esophageal endoscopy were not performed in every patient, although there are data indicating a comprehensive screening for selected SSc-specific complications [38–40]. Another limitation may be that parameters of endothelial dysfunction were only measured at baseline visit, but not during subsequent annual study visit. Potentially relevant changes of parameters of endothelial dysfunction were therefore overlooked. Moreover, some lcSSc patients had concomitant cardiovascular risk factors which may also affect parameters of endothelial dysfunction.

In conclusion, endothelial dysfunction may be a slight and potential trigger for development of SSc-specific complications in patients with lcSSc, although its predictive value needs to be more thoroughly evaluated in larger and long-term prospective studies.

## Electronic supplementary material

Below is the link to the electronic supplementary material.

Supplementary Material 1: Changes of telangiectasia (a), sclerodactyly (b), puffy finger (c), early (d), active (e) and late pattern (f) during follow-up period.

Supplementary Material 2: Changes of CRP (a), eGFR (b), protein/creatinine ratio (c), predicted DLCO (d), FEV1/FVC (e) and predicted TLC (f) during follow-up period.

## Data Availability

The data that support the findings of this study are available from the corresponding author upon reasonable request.

## Abbreviations

ADMA: Asymmetric dimethylarginine
Aix: Augmentation index
CAD: Coronary heart disease
CRP: C-reactive protein
CSURI: Capillaroscopic skin ulcer risk index
CVAD: Carotid and vertebral artery disease
DLCO: Diffusing capacity of the lung for carbon monoxide
DU: Digital ulcer
eGFR: Estimated glomerular filtration rate
EMP: Endothelial microparticles
FMD: Flow-mediated dilation
ILD: Interstitial lung disease
lcSSc: Limited cutaneous systemic sclerosis
mRSS: Modified Rodnan Skin Score
NMD: Nitroglycerine-mediated dilation
NT-proBNP: N-terminal prohormone of brain natriuretic peptide
PAD: Peripheral artery disease
PH: Pulmonary hypertension
PWV: Pulse-wave velocity
SDMA: Symmetric dimethylarginine
SSc: Systemic sclerosis
TLC: Total lung capacity
vWF: von Willebrand factor
vWF:Ac: von Willebrand factor activity
vWF: Ag: von Willebrand factor antigen
