# Supplementary figures and images for: Endothelial function and vascular events in patients with limited cutaneous systemic sclerosis (EFVELSS): a prospective observational study
Source: Rheumatol Int. 2025 Jul 8;45(7):166. doi: 10.1007/s00296-025-05919-y (PMC12238130; doi:10.1007/s00296-025-05919-y)

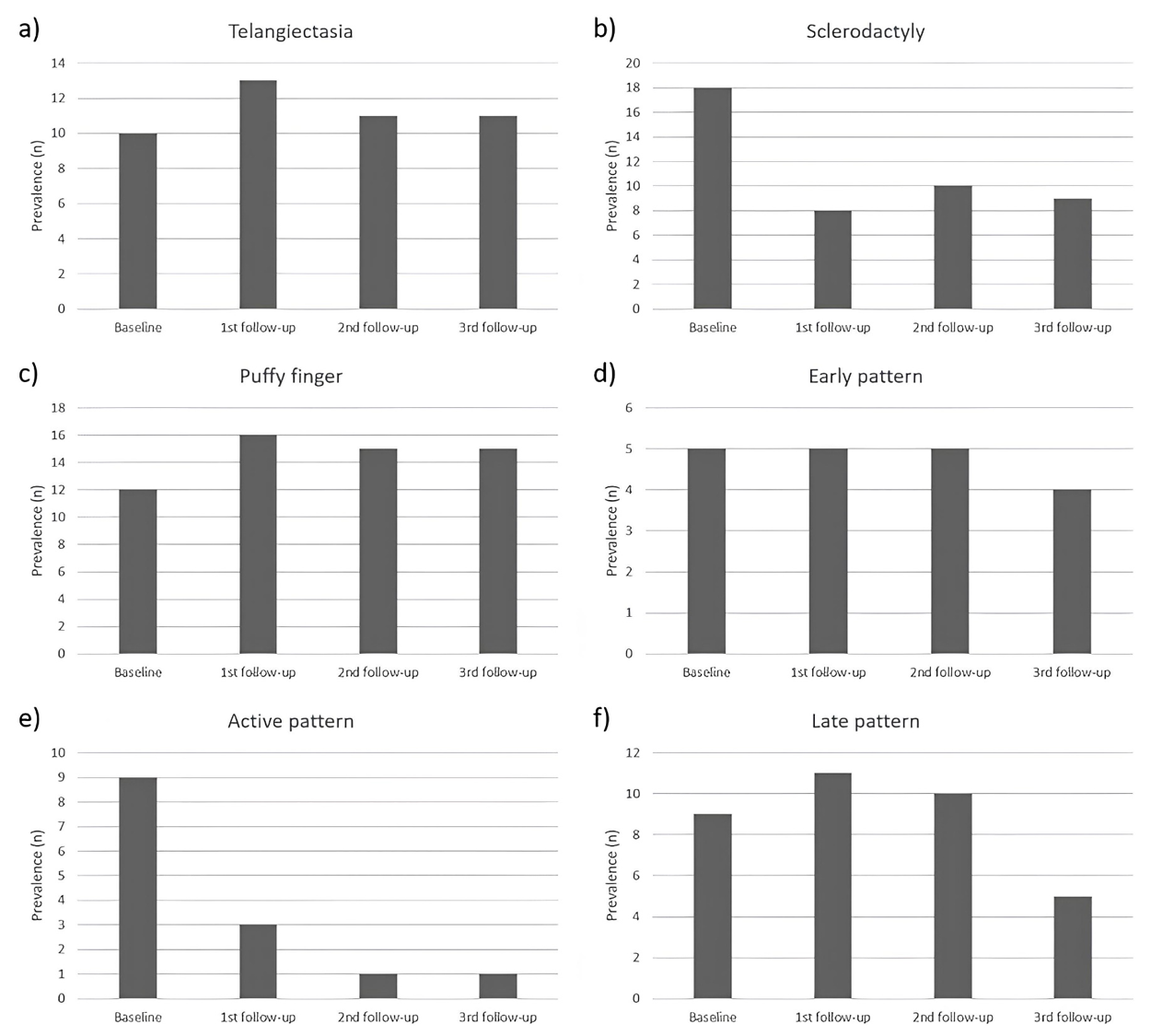

Supplement: Supplementary file 1 — Supplementary Material 1: Changes of telangiectasia (a), sclerodactyly (b), puffy finger (c), early (d), active (e) and late pattern (f) during follow-up period. [file 296_2025_5919_MOESM1_ESM.tif]

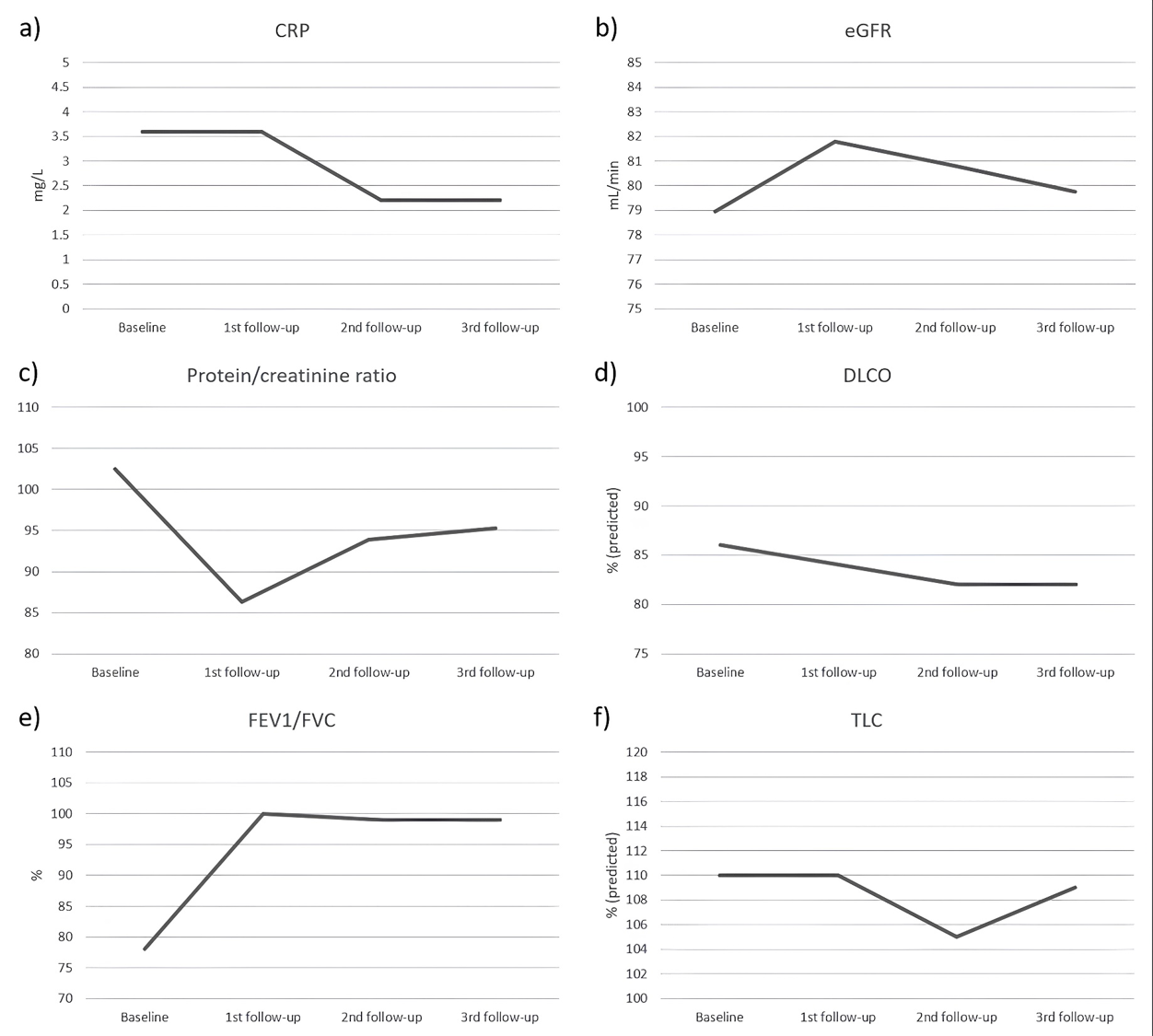

Supplement: Supplementary file 2 — Supplementary Material 2: Changes of CRP (a), eGFR (b), protein/creatinine ratio (c), predicted DLCO (d), FEV1/FVC (e) and predicted TLC (f) during follow-up period. [file 296_2025_5919_MOESM2_ESM.tif]
